# In utero exposure to thermal stress has long-term effects on mammary gland microstructure and function in dairy cattle

**DOI:** 10.1371/journal.pone.0206046

**Published:** 2018-10-16

**Authors:** Amy L. Skibiel, Bethany Dado-Senn, Thiago F. Fabris, Geoffrey E. Dahl, Jimena Laporta

**Affiliations:** Department of Animal Sciences, University of Florida, Gainesville, FL, United States of America; University of Illinois, UNITED STATES

## Abstract

Earth’s rising temperature has substantial repercussions for food-producing animals by increasing morbidity and mortality, diminishing reproductive potential, and reducing productivity. In the dairy industry this equates to massive losses in milk yield, which occur when cows are exposed to heat stress during lactation or during the non-lactating period between lactations (i.e. dry period). Furthermore, milk yield is significantly lower in first-lactation heifers that experienced fetal heat stress. The mechanisms underlying intrauterine effects of heat stress on the offspring’s future lactation have yet to be fully elucidated. We hypothesize that heat stress experienced through the intrauterine environment will alter the mammary gland microstructure and cellular processes involved in cell turnover during the cow’s first lactation. Mammary biopsies were collected from first-lactation heifers that were exposed to heat stress or cooling conditions while developing in utero (IUHT and IUCL; respectively, *n* = 9–10). IUHT heifers produced less milk compared to IUCL. The mammary glands of IUHT heifers differed morphologically from IUCL, with the IUHT heifers having smaller alveoli and a greater proportion of connective tissue relative to their IUCL herdmates. However, intrauterine heat stress had little impact on the proliferation and apoptosis of mammary cells during lactation. Our results indicate that fetal exposure to heat stress impairs milk production in the first lactation, in part, by inducing aberrant mammary morphology. This may result from alterations in the developmental trajectory of the fetal mammary gland that persist through the first lactation rather than to alterations in the cellular processes controlling mammary cell turnover during lactation.

## Introduction

Environmental heat stress negatively impacts livestock health, welfare, and productivity [1]. High producing dairy cattle are especially susceptible to heat stress. A temperature-humidity index above 68 can induce physiological and behavioral responses that prioritize thermoregulation over other physiological processes, such as milk synthesis [2–4]. In the United States alone, approximately 90% of the dairy cow population resides in states with at least 55 heat stress days per year, resulting in enormous milk yield losses [5]. Although cows are vulnerable to heat stress during lactation, environmental heat stress during the dry period also adversely affects cow physiology and milk production in the subsequent lactation [6–8].

In dairy cattle, the dry period, a six to eight-week non-lactating state between lactations, coincides with late gestation, an important stage of fetal growth and development. During the last two months of gestation, the fetus grows at the fastest rate and accumulates approximately 60% of its birth weight [9]. Calves born to dams that were heat stressed when dry are lighter from birth to one year of age and have compromised immune function [6, 10, 11]. Furthermore, these female calves (i.e. heifers) produce less milk through their first lactation relative to heifers born to cooled dams [12], suggesting a permanent effect of the fetal environment on lactation phenotype. Although the most extensive mammary growth occurs during pregnancy, mammary morphogenesis begins in the embryo and a rudimentary ductal system with minimal secretory epithelium exists at birth [13]. Perturbations in the intrauterine environment affecting normal embryonic mammogenesis may have long-term consequences for the future development and function of the mammary gland [14]. There is evidence in sheep that maternal effects on mammary ductal development in the fetus impact the offspring’s lactation performance later in life [15]. However, the mechanisms mediating effects of intrauterine heat stress on mammary function are unknown.

The capacity of the mammary gland to synthesize and store milk depends on the maintenance of both the number and activity of secretory mammary epithelial cells (MEC) throughout lactation and the size of the alveoli, the functional units of the mammary gland [16–18]. The relative rates of apoptosis (i.e. programmed cell death) and cell proliferation (i.e. cell division and cell growth) determine the number of MEC in the mammary gland [18]. MEC number gradually declines throughout lactation as apoptotic rate exceeds the rate of cell proliferation; however, substantial cell turnover occurs such that at least 50% of the MEC present in the mammary gland at the end of lactation form after calving [19]. Thus, the dynamic relationship between apoptosis and cell proliferation is critical to mammary function during lactation. Apoptosis and cell proliferation are both affected by heat stress. Subjecting bovine MEC in vitro to acute thermal stress results in the downregulation of genes involved in cell morphogenesis and cytoskeletal structure and upregulation of pro-apoptotic genes [20, 21]. Furthermore, acute heat stress represses MEC proliferation and ductal branching whereas the proportion of apoptotic cells is elevated [20, 22]. In vivo, heat stress decreases cell proliferation in the late dry period of Holstein dairy cows [7]. Together these studies point to altered cellular processes in the mammary gland of heat stressed cows that may be responsible for poor lactation performance.

The objective of the current study was to evaluate the microstructure and cellular processes of the mammary gland of first-lactation heifers born to dams heat stressed during the dry period (i.e. in late gestation). We predicted that in utero exposure to heat stress would alter mammary gland architecture and cellular processes through the first lactation, thus impairing milk synthesis.

## Materials and methods

### Animals and treatments

All procedures performed in this study were approved by the Institutional Animal Care and Use Committee at the University of Florida.

#### Dams and heifers

In 2014 and 2015, 51 multiparous dams were either heat stressed (2014, *n* = 10; 2015, *n* = 18) or cooled (2014, *n* = 10; 2015, *n* = 13) during their dry period (i.e. last 46 d of gestation) as described in detail in Monteiro et al. [23] and Fabris et al. [8]. Briefly, multiparous pregnant dry cows were housed in a shaded, sand-bedded, freestall barn either with active water soakers and fans (cooled) or with inactive soakers and fans (heat stressed). On the cooled side of the barn, fans ran continuously and water soakers turned on for 1.5 min in 5 min intervals when the ambient temperature rose above 21.1°C. Heifer calves born to these dams experienced heat or cooling conditions through the intrauterine environment (IUHT or IUCL, respectively) and were used in this study. After birth, heifers were separated from their dams and had their navels dipped prophylactically in 2% iodine. Colostrum from all dams, irrespective of treatment, was pooled and fed to heifers in two meals; 3.8 L within 4 h of birth and another 2.8 L within 12 h of birth. Thereafter, 1.9 L of pasteurized milk was fed twice a day (0600 and 1400 h) up to 29 d and then 3.8 L per feeding to 41 d. Calves were gradually weaned from 42 to 49 d by feeding 1.9 L in the morning only. Throughout the pre-weaning period, starter grain was provided ad libitum. Heifers were kept in individual hutches from birth to 10 d after weaning, housed in group pens with 8 to 10 heifers until 129 d post-weaning, and then moved to larger pens of 18 to 20 heifers. Management and environmental conditions were identical for all heifers from birth through their first lactation. First calving of the heifers occurred from May to September in both years. Out of the 20 heifers born in 2014, 3 IUHT and 8 IUCL calved and began lactating in 2016. Out of the 31 heifers born in 2015, 14 IUHT and 10 IUCL calved and began lactating in 2017. Heifers left the herd before calving for various reasons (e.g. culled/sold for health, growth, or reproductive reasons, or died). Post-calving, all heifers were housed in shaded, freestall barns with operating water soakers and fans and fed a total mixed ration twice a day. Cows were milked 4 times a day for the first 21 days in milk (DIM) and twice daily thereafter.

### Milk yield and physiological measures

Respiration rate and rectal temperature were recorded at 1400 h every 7 days post-calving until 42 DIM. Colostrum yield (i.e. yield at first milking) and daily milk yield up to 84 DIM were compiled from AfiFarm Dairy Herd Management Software (Afimilk Ltd., Kibbutz, Afikim, Israel). Gestation length was calculated as the artificial insemination date subtracted from the calving date. Calves born to IUHT and IUCL heifers were weighed by farm staff at birth prior to colostrum feeding.

### Mammary biopsies and tissue processing

Mammary biopsies were collected on 21 and 42 DIM for a subset of IUHT (*n* = 9) and IUCL heifers (*n* = 10) in 2016 and 2017. Biopsies were performed following the method described by Farr et al. [24], with modifications by our group [7]. Biopsies were taken from the right or left rear quarter of the mammary gland, alternating sides for each subsequent biopsy. Cows were anesthetized with an intravenous injection of xylazine (Akom Inc., Decatur, IL) at a concentration of 20 mg/ml and a dose of 35 μl/kg of body weight. A small region of the udder was shaved and scrubbed with iodine and isopropanol to sanitize the biopsy field, followed by subcutaneous administration of 3 ml of lidocaine in a line block above the biopsy site for local anesthesia. A 3 to 4 cm incision was made with a scalpel blade through the skin and capsule of the udder. A core of mammary tissue was extracted using a stainless steel biopsy tool attached to a drill. Following biopsy retrieval, the incision area was closed with Michel 18 mm wound clips and antiseptic spray was applied. Biopsied tissue was rinsed in PBS, trimmed of fat, and a 5 mm piece was cut for histology. After fixing in 4% paraformaldehyde at 4°C overnight, the tissue was rinsed in increasing concentrations of ethanol (25%, 50%, 70%, and 100%), and then transferred to 70% ethanol and stored at 4°C until embedding. Tissues were processed, embedded in paraffin, and sectioned at 5 μm onto poly-L-lysine coated slides. Sectioned tissues were stained with hematoxylin and eosin (H&E), stained with Masson’s trichrome stain, or used for immunohistochemistry. For both years, embedding, sectioning, and staining was completed within 2 months of sample collection.

### H&E and Masson’s trichrome staining

Histological staining with hematoxylin and eosin was performed to visualize the tissue microstructure, allowing for cells and structures (e.g. mammary epithelial cells and mammary gland alveoli) to be quantified. Masson’s trichrome stain was performed to visualize the connective tissue. Both techniques were performed following standard protocols at the Molecular Pathology Core at the University of Florida.

### Immunohistochemistry

#### Ki67 assay for cell proliferation

Slides were incubated at 60°C for 30 minutes, deparaffinized in xylene, and rehydrated in a graded series of ethanol washes (100%, 95%, 70%) followed by a rinse in distilled-deionized water. The UltraVision One Detection System: HRP Polymer/DAB Plus Chromogen kit (Thermo Fisher, cat. # TL-015-HDJ) was used for the Ki67 assay. Antigen retrieval was achieved by incubating slides in a coplin jar with 1 x citrate buffer in a water bath at 95°C for 20 min. Endogenous peroxidases were blocked by quenching slides in H_2_O_2_. After washing in 1 x TBS with 0.1% Tween-20, tissue sections were blocked with the UltraV block provided in the kit for 5 min. Sections were then incubated with primary antibody (Monoclonal Mouse Anti-Human Ki67 Antigen Clone MIB-1, Dako Agilent, cat. # M724029-2) diluted 1:100 in 1 x TBS for 30 min at room temperature. Sections were incubated with UltraVision One HRP Polymer from the kit for 30 min at room temperature, subsequently washed in 1 x TBS with 0.1% Tween-20, and incubated for 5 min with 3,3’-diaminobenzidine substrate from the kit, diluted according to the manufacturer’s recommendation. Sections were counterstained with hematoxylin, subjected to a series of graded ethanol washes and xylene, and mounted with Permount (Thermo Fisher, cat. # SP15). Mammary tissue of a cow at 14 DIM (from a separate study) was run without primary antibody as a negative control. Jejunum tissue from a 2-day old calf (from a separate study), expected to have a high proliferation rate, was used as a positive control.

#### Tdt dUTP nick-end labeling (TUNEL) assay for apoptosis

ApopTag Plus Peroxidase *in situ* Apoptosis Kit (Millipore, cat. # S7101) was used following the manufacturer’s protocol with a few modifications. Tissue sections were first deparaffinized in xylene followed by a graded series of ethanol washes for rehydration and a rinse in 1 x PBS. Antigen retrieval was conducted via the enzymatic method, incubating sections in 20 μg/ml proteinase K (Invitrogen, cat. # AM2542) for 8 min at room temperature. After several washes in distilled-deionized water, tissue sections were quenched in 3% hydrogen peroxide in 1 x PBS for 10 min, and incubated for 60 min at 37°C with TdT enzyme. Sections were then incubated with anti-digoxigenin-peroxidase for 30 min at room temperature followed by 3,3’-diaminobenzidine substrate for 8 min. Methyl green was used for the counterstain and tissue sections were dehydrated with n-butanol and xylene, then mounted with Permount. Mammary tissue of a cow 14 DIM was run with TdT enzyme diluted in stop/wash buffer instead of reaction buffer as a negative control. The Apoptag kit contains slides of involuting rat mammary gland collected 3–5 days post-weaning, which was used as a positive control.

### Quantification of histological sections

Histological slides were imaged with an EVOS XL Core imaging system (Advanced Microscopy Group, Bothell, WA). For H&E and trichrome stained sections, 5 photomicrographs were captured per section with a 20X objective for quantification of alveoli number and luminal area. Alveoli were defined as any rounded structure with a lumen enclosed by a single layer of epithelial cells. Alveoli area was measured as the luminal space, not including the area comprised by the surrounding mammary epithelial cells. Ductal structures were not included in counts or area measurements. Fields were selected at random but areas close to the edge of the section were avoided. Alveoli area, connective tissue area, and the number of alveoli and stained cells were quantified using ImageJ [25]. For count data, the point picker plugin [26] in ImageJ was used. Area measurements were calibrated to the image size and magnification (i.e. pixels to μm). Total tissue area for photomicrographs taken at 20X is 574,497 μm^2^ and at 40X is 96,820 μm^2^. For H&E and trichrome stained slides, alveoli number, alveoli area, and connective tissue area, respectively, were averaged across the 5 fields measured per section and the averages were used for statistical analyses. Percent of connective tissue in the mammary gland was calculated as connective tissue area divided by total tissue area at 20X, multiplied by 100. To describe the relationship between alveoli area and number of secretory cells, alveoli area was measured and secretory cells were counted across 5 photomicrographs per section taken with a 40X objective from 21 DIM samples.

For Ki67 and TUNEL labeled tissue sections, 5 photomicrographs were taken per section using a 40X objective. For Ki67 and TUNEL, every cell was counted in each of the 5 fields per section and scored as positive (stained brown, indicating cell undergoing proliferation or apoptosis) or negative (stained blue, indicating the cell was not undergoing proliferation or apoptosis). This resulted in a total of more than 3,000 cells counted per section. All cells were classified as either a mammary epithelial cell or a stromal cell. Mammary epithelial cells (MEC) included mammary alveolar cells, ductal epithelial cells, and myoepithelial cells. Stromal cells were considered to be the cells in the area surrounding the epithelium, such as fibroblasts, endothelial cells, blood cells, immune cells, and adipocytes as described in Tao et al. [7]. The proportion of cells proliferating was calculated as the total number of positive cells across all 5 fields within each cell type (MEC or stromal) divided by the total number of cells (positive plus negative) within the same cell type (i.e. proliferating MEC = positive MEC/total MEC, proliferating stromal cells = positive stromal cells/total stromal cells). Proportion of cells undergoing apoptosis was calculated in the same manner.

### Statistical analyses

All data were statistically analyzed using SAS v. 9.4 (SAS Institute, Cary, NC). Colostrum yield, gestation length, and calf birth weight were analyzed by one-way ANOVA (PROC GLM). The model for calf weight included calf sex as a covariate and an interaction between sex and treatment. Models for colostrum yield and gestation length included year as a covariate and an interaction between year and treatment. Correlations between alveoli luminal area and number of secretory cells comprising the alveoli were assessed by Pearson correlation (PROC CORR). Proliferation and apoptosis data were analyzed by generalized linear mixed models (PROC GLIMMIX) with a binomial distribution, logit link function, year, treatment, and DIM as fixed effects, and heifer ID as a random effect. All other variables were analyzed by general linear mixed models (PROC MIXED) with treatment, DIM, year, and interactions as fixed effects, DIM within subject ID as the repeated measure, and compound symmetric or autoregressive covariance structure chosen based on AIC. Data were checked for normality and outliers. A *P*-value less than or equal to 0.05 was considered significant. *P*-values less than 0.1 but greater than 0.05 were considered a tendency. Data are presented as least squares means (LSM) ± standard error of the mean (SEM) unless indicated otherwise.

## Results

### Milk yield and physiological measures

The dams of the heifers had significantly lower respiration rates and rectal temperatures if provided with active cooling during their dry period (i.e. during the last 46 d of gestation). THI of the heat and cooled sides of the barn were similar in 2014 and 2015 across the entire dry period (average was 77.75 [8, 23]). Thus, dams in the cooled group experienced heat abatement and heifers born to heat stressed or cooled dams experienced different intrauterine environments.

When heifers born to heat or cooled dams were evaluated at maturity, there were no differences in their rectal temperature or respiration rate during the first 42 DIM (Table 1). However, for rectal temperature there was a significant interaction between treatment and year (*P* = 0.003), where IUHT had significantly lower rectal temperatures in 2016, but higher temperatures in 2017 compared to IUCL. IUHT and IUCL heifers had similar gestation lengths, similar body weights throughout lactation, and their calves were similar in body weight at birth (Table 1). IUHT cows had higher colostrum yield but lower milk yield than IUCL cows (*P* < 0.0001 and *P* = 0.05, respectively). Milk yield increased over the course of lactation (*P* < 0.0001) and there was a significant year effect (*P* = 0.01), where heifers produced, on average, 2 kg/d more milk in 2016 than 2017.

**Table 1:** Physiological and production measures of first-lactation heifers that were exposed to heat stress (IUHT, *n* = 17) or cooling (IUCL, *n* = 18) conditions through the intrauterine environment.

	Treatment		* *
Variable	IUHT	IUCL	*P-*value
Rectal temperature (°C)	39.1 ± 0.1	39.0 ± 0.1	0.48
Respiration rate (breaths/min)	65.9 ± 2.5	65.2 ± 2.3	0.83
Calf weight (kg)	37.6 ± 1.2	39.5 ± 1.4	0.68
Heifer weight (kg)	533.5 ± 5.00	539.5 ± 4.7	0.37
Gestation length (d)	275.6 ± 1.3	274.8 ± 1.0	0.65
Colostrum yield (kg)	5.5 ± 0.44	3.7 ± 0.5	**<0.0001**
Milk yield (kg/day)	30.2 ± 0.5	31.5 ± 0.4	**0.05**

Statistical analyses are general linear mixed models with treatment (IUHT, IUCL), days in milk (DIM), year, and interactions as fixed effects and DIM within subject ID as a repeated measure. *P*-value is for the main effect of treatment. Rectal temperature and respiration rate were recorded from a subset of heifers (*n* = 9 for IUHT and *n* = 10 for IUCL). Heifer weight = average weight across 84 DIM. There was an interaction between treatment and year on rectal temperature (*P* = 0.003). No other interactions were significant. Milk yield increased through the first 84 DIM (*P* < 0.0001) and there was a significant year effect for milk yield (*P* = 0.01). Data are presented as LSM ± SEM. Bold-faced font indicates significant difference at *P* ≤ 0.05.

### Mammary gland microstructure

**Fig 1:**
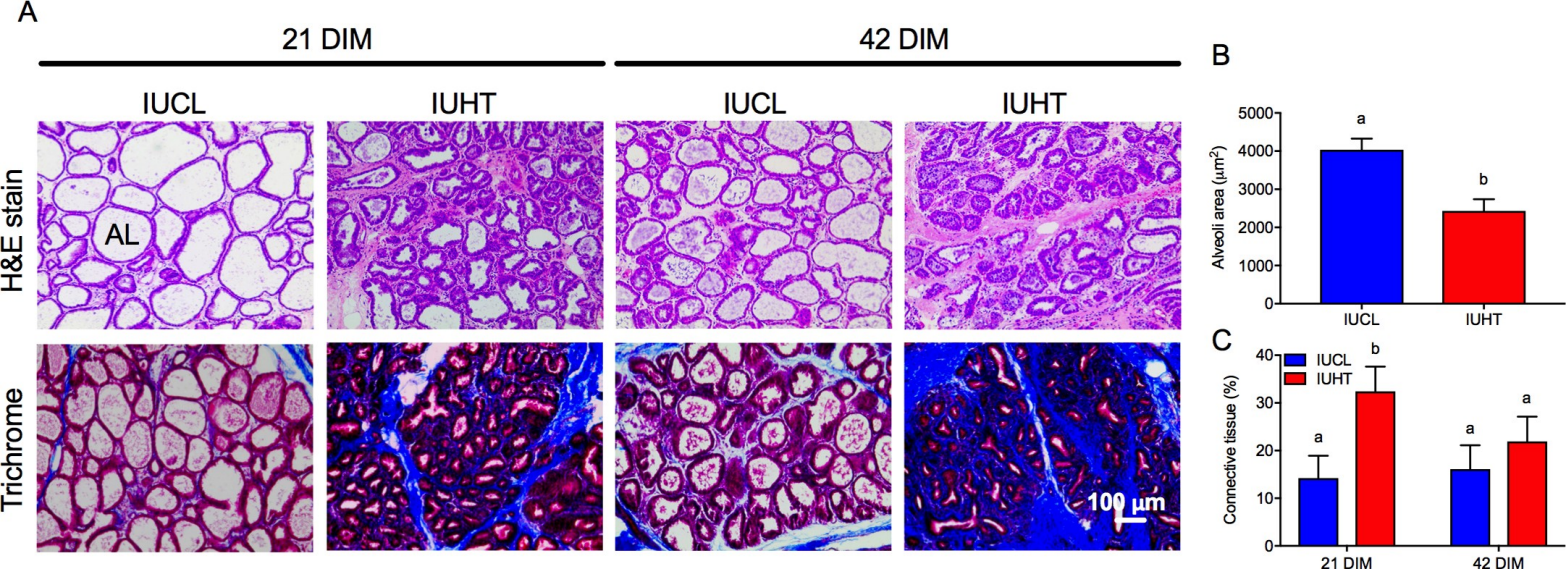
Histological evaluation of the mammary gland from first-lactation heifers. Heifers were born to dams actively cooled with fans and water soakers (in utero cooled, IUCL; *n* = 10) or heat stressed (no heat abatement, in utero heat stressed, IUHT; *n* = 9) during the last 46 d of gestation. Mammary biopsies were taken from heifers at 21 and 42 days in milk (DIM). (A) Hematoxylin and eosin (H&E) stained and Masson’s trichrome stained mammary tissue at 20X. Connective tissue is stained blue. (B) Luminal area of mammary alveoli was smaller for IUHT (red bars) compared to IUCL (blue bars) (*P* = 0.001). (C) IUHT had a higher percent connective tissue comprising the mammary gland at 21 DIM compared to IUCL (*P* = 0.03). Total tissue area at 20X is 574,497 μm^2^. Data presented as LSM ± SEM. Disparate letters indicate significant differences. AL = alveoli lumen.

**Fig 2:**
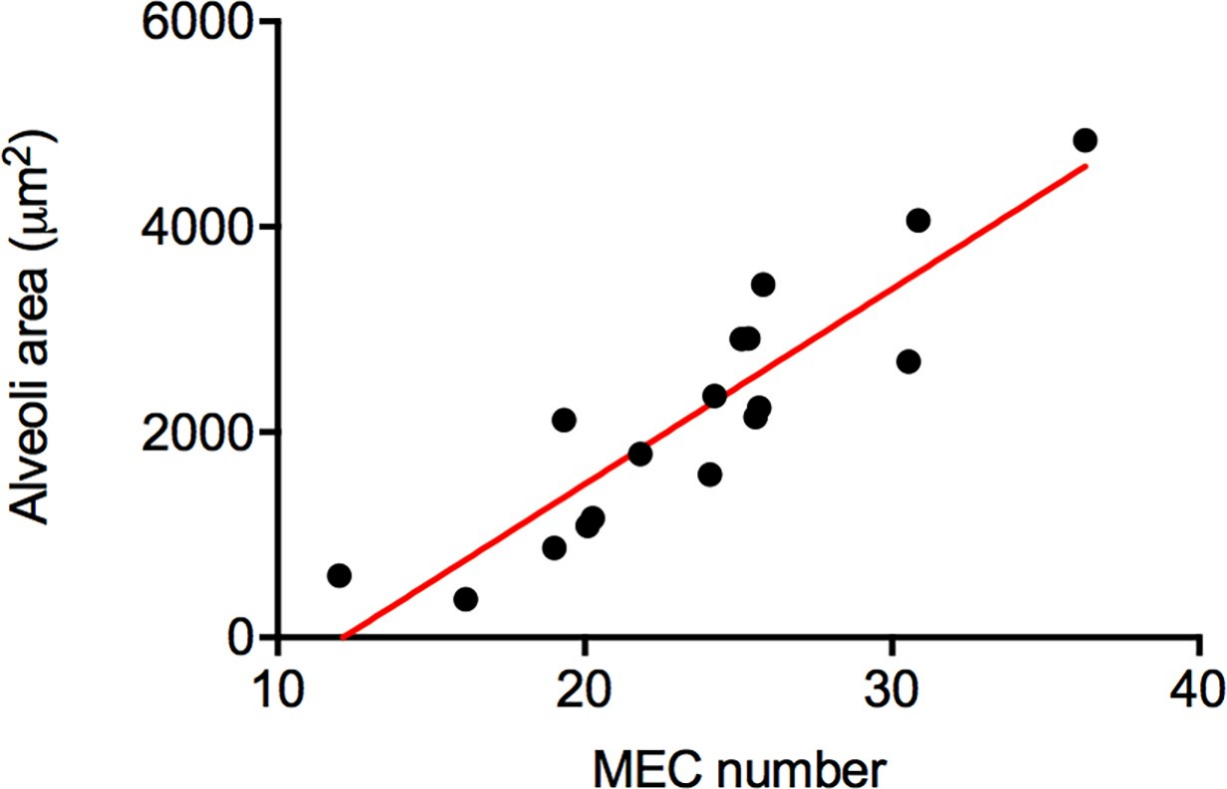
Correlation between luminal area of mammary alveoli and mammary epithelial cell number. Data are from mammary tissue collected from lactating cows at 21 days in milk (DIM). Data presented as mean ± SEM for in utero heat and cooled cows combined, r = 0.90, *P* < 0.001.

The mammary tissue of heifers had a similar number of alveoli (IUHT: 54.8 ± 2.8, IUCL: 51.5 ± 2.6, *P* = 0.39). However, the alveoli from IUHT were significantly smaller in area compared to alveoli from the IUCL heifers (2431.6 ± 307.8 vs. 4037.0 ± 288.6, *P* = 0.001; Fig 1). Alveoli area was larger in 2016 than 2017 regardless of treatment (*P* = 0.003). For both treatment groups, there were more alveoli present in the mammary gland at 21 DIM compared with 42 DIM (21 DIM: 58 ± 2.6, 42 DIM: 48 ± 2.7, *P* = 0.01). Interaction terms were not significant for alveoli area or number. Alveoli luminal area was positively correlated with the number of secretory cells comprising them (*r* = 0.90, *P* < 0.001; Fig 2). Results were consistent when data from IUHT and IUCL were run separately, thus the correlation presented herein contains data pooled across both groups.

There was a significant treatment by DIM interaction on percent connective tissue in the stroma of the mammary gland (*P* = 0.03, Fig 1). Mammary glands of IUHT heifers had a significantly higher percent connective tissue relative to IUCL at 21 DIM. IUHT heifers had a higher percent connective tissue at 21 DIM versus 42 DIM whereas percent connective tissue for IUCL was similar between 21 DIM and 42 DIM (Fig 1).

### Cell proliferation and apoptosis

**Fig 3:**
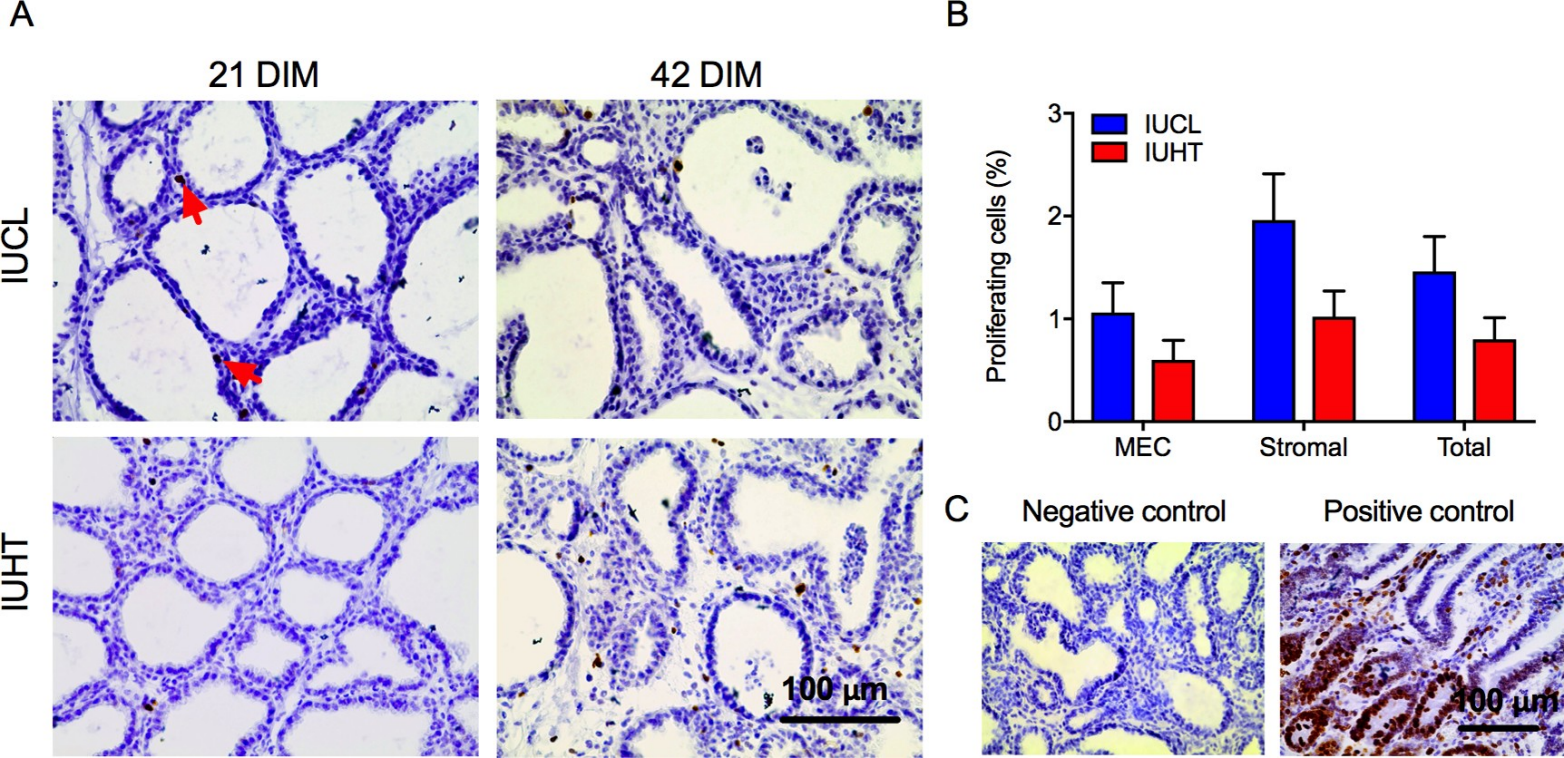
Immunohistochemistry of proliferating (Ki67) cells in the mammary gland of first-lactation heifers. Heifers were born to dams actively cooled with fans and water soakers (in utero cooled, IUCL; *n* = 10) or heat stressed (no heat abatement, in utero heat stress, IUHT; *n* = 9) during the last 46 d of gestation. Mammary biopsies from heifers were collected at 21 and 42 days in milk (DIM). (A) Ki67 staining of cells in mammary tissue. (B) Percent of proliferating mammary epithelial cells (MEC), stromal cells, and total mammary cells. There was a tendency for IUHT heifers (red bars) to have a lower percent of proliferating mammary cells compared to IUCL heifers (blue bars) (*P* = 0.09). (C) Negative (14 DIM mammary tissue without primary antibody) and positive (jejunum tissue from a 2-day old calf) controls for the Ki67 assay. Data are presented as LSM ± SEM. Red arrows indicate proliferating cells (stained brown).

**Fig 4:**
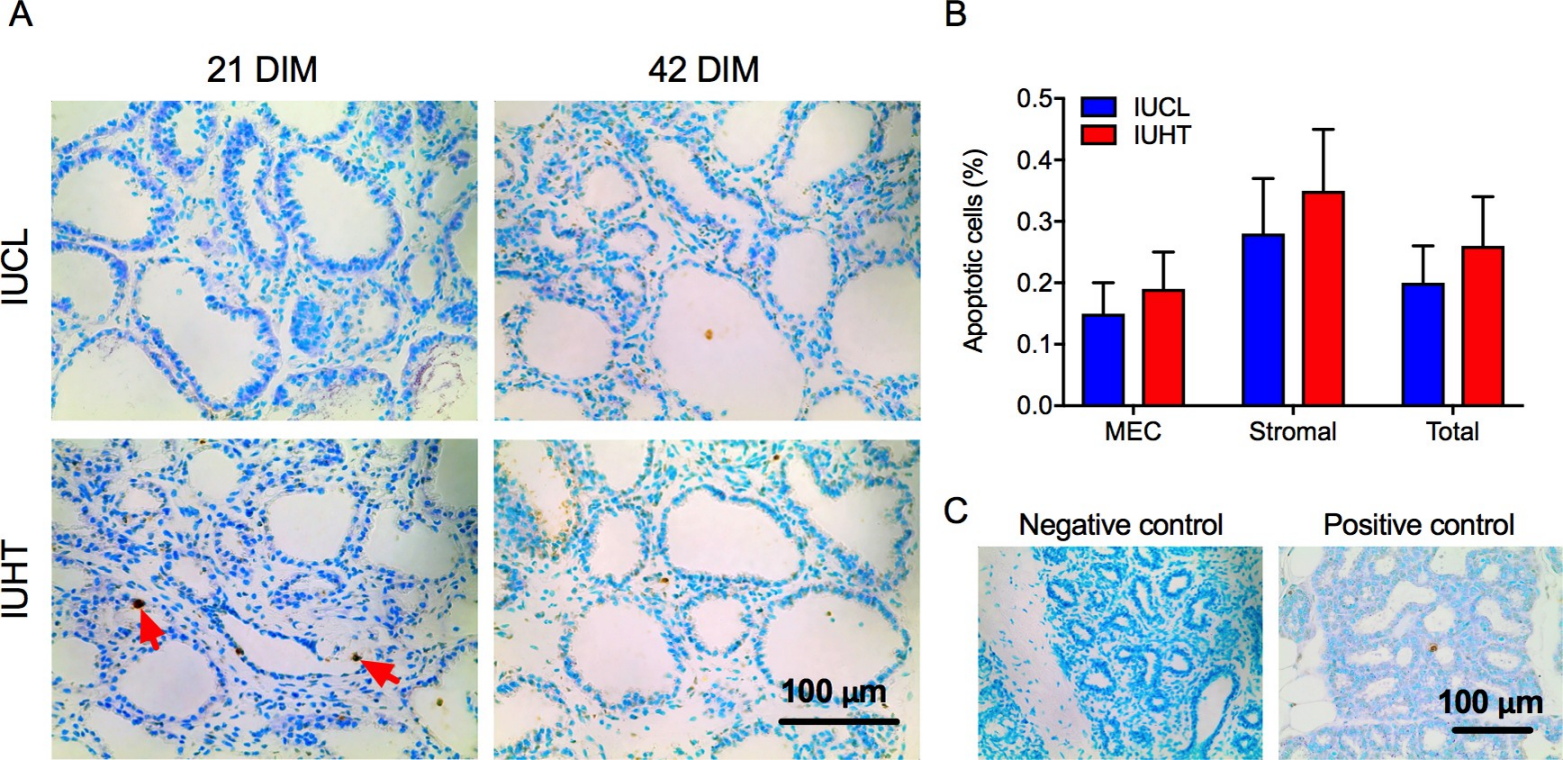
Immunohistochemistry of apoptotic (TUNEL) cells in the mammary gland of first-lactation heifers. Heifers were born to dams actively cooled with fans and water soakers (in utero cooled, IUCL; *n* = 10) or heat stressed (no heat abatement, in utero heat stress, IUHT; *n* = 9) during the last 46 d of gestation. Mammary biopsies were collected at 21 and 42 days in milk (DIM). (A) TUNEL staining of cells in mammary tissue. (B) Percent of mammary epithelial cells (MEC), stromal cells, and total mammary cells undergoing apoptosis. (C) Negative and positive controls for the TUNEL assay. The negative control was mammary tissue of a 14 DIM cow run with TdT enzyme diluted in stop/wash buffer instead of reaction buffer. The positive control was rat mammary gland 3–5 days post-weaning. Differences in the percent of apoptotic cells between IUHT (red bars) and IUCL (blue bars) heifers were not significant for any cell type (*P* > 0.05). Data are presented as LSM ± SEM. Red arrows denote cells undergoing apoptosis (stained brown).

There was a tendency for IUHT heifers to have a lower percent of proliferating cells in the mammary gland compared to IUCL (0.8 ± 0.2% vs. 1.5 ± 0.3%, *P* = 0.09). This tendency was largely attributed to a lower percent of proliferating cells in the stromal compartment (*P* = 0.06; Fig 3). Exposure to heat stress in utero did not have an effect on the percent of cells undergoing apoptosis as IUHT and IUCL had a similar percent of mammary epithelial and stromal cells undergoing apoptosis (*P* > 0.52, Fig 4).

## Discussion

Maternal heat stress exposure during late gestation reduces milk yield during the first lactation of their offspring [12]. However, the biological mechanisms linking intrauterine conditions to lactation performance later in life was hitherto unknown. Herein, we show that intrauterine heat stress impacts the microstructure and cellular processes in the mammary gland of heifers during their first lactation, 2 years after heat exposure occurred. During their first lactation, IUHT heifers had lower rectal temperatures than IUCL heifers, but only in 2016. This result is consistent with a recent study whereby IUHT cows had lower body temperatures and respiration rates relative to IUCL cows after both groups were subjected to a 48 hr heat challenge midway through their first lactation [27].

IUHT heifers had higher colostrum yield but produced 1.3 kg/d less milk through the first 84 days in milk relative to IUCL heifers. A compilation of production data from 5 years of studies employing an experimental design identical to the present study revealed that IUCL heifers produced 5.1 kg more milk per day in the first lactation relative to IUHT heifers [12]. This difference in milk yield occurred even though both groups were similar in body mass around the time of parturition and through lactation, implying that body size at calving did not contribute to differences in lactation performance. Similarly, in the present study, differences in milk yield were not attributed to differences in heifer body weight during lactation, calf birth weight, or to differences in the length of time available for mammary growth during gestation as both groups had similar gestation lengths.

Milk yield is a function of the number of secretory cells, the metabolic activity of those cells, and the size of the alveoli formed by the secretory cells [13, 18, 19]. Secreted milk is stored in the alveoli and ducts of the gland until milk ejection; thus, mammary storage capacity, and hence the volume of milk removed, is dependent, in part, on the luminal area of the alveoli comprising the gland [28, 29]. In fact, the volume of milk stored in the mammary alveoli of peak and late lactation cows, up to 12 h post-milking, is greater than the amount of milk stored in the gland cistern [30]. In our study, IUHT heifers had a similar number of mammary alveoli, but alveoli were 46% smaller than IUCL heifers. Alveoli with smaller luminal area will have lower capacities for milk storage and may also have a lower capacity for milk synthesis because they contain fewer secretory cells, as indicated by the positive correlation between alveoli luminal area and the number of secretory cells they contain. Thus, the reduced size of mammary alveoli among IUHT heifers may be one of the contributing factors to their reduced lactation performance relative to IUCL heifers.

Cell proliferation and apoptosis, the main cellular processes involved in mammary remodeling, together determine the number of cells in the mammary gland throughout lactation [18]. The gradual rise in milk yield from early to peak lactation is attributed to greater secretory activity and functional differentiation of MEC, whereas late lactation is typified by a higher rate of apoptosis relative to cell proliferation [19]. Although proliferation of MEC is relatively low throughout lactation, the dynamic balance between cell proliferation and apoptosis across lactation results in substantial cell turnover within the mammary gland during this stage of the production cycle [18, 19, 31]. Both cell proliferation and apoptosis are critical for optimizing mammary function by maintaining cell numbers essential for lactation. These cellular processes are also affected by high ambient temperatures [20, 32, 33]. Genes involved in cytoskeletal structure and cell biogenesis are downregulated whereas genes associated with apoptosis are upregulated in bovine mammary epithelial cell lines following acute exposure to high temperature [20, 21]. In addition, ductal branching is repressed, cell proliferation is inhibited, cell viability is impaired, and a greater proportion of apoptotic cells are observed after acute heat stress [20–22]. Thus, even mild acute heat stress is sufficient to induce apoptosis and inhibit growth of mammary cells. In our in vivo study where heifers were exposed to heat stress or cooling conditions through the intrauterine environment, there was a tendency for IUHT heifers to have a lower percent of proliferating mammary cells relative to IUCL heifers. Likewise, multiparous cows cooled during the dry period had a greater rate of MEC proliferation compared to heat stressed cows [7]. We found no significant differences between IUHT and IUCL heifers in the percent of mammary cells undergoing apoptosis. Similarly, the apoptotic rate of the mammary gland was not influenced by heat stress during the dry period among multiparous cows [7]. However, besides apoptosis, other cell processes, such as autophagy, are involved in MEC removal from the mammary gland and are altered by heat stress [34] and therefore warrant further exploration.

Mammary development begins in utero. At birth, teats and a rudimentary ductal compartment are present, although the secretory tissue will not form extensively until the first pregnancy [13, 35]. Milk production is positively correlated with the amount of secretory tissue comprising the mammary gland, which develops from the terminal ends of the ducts [36]. Thus, it is possible that intrauterine conditions affecting ductal growth may have consequences for future secretory tissue development and lactation performance. Maternal size and nutrition during gestation impact mammary development of the fetus through postnatal life [15, 37, 38], For example, ewes fed a maintenance diet versus ad libitum had fetuses with heavier mammary glands at 100 days of fetal age and heavier ewes had fetuses with greater mammary ductal area. Moreover, differences in fetal mammogenesis had ramifications for future lactation performance; progeny born to maintenance-fed and heavier ewes had greater milk yields during their first lactation [15]. The specific mechanisms through which in utero conditions permanently impact adult lactation are poorly understood, although an intriguing possibility is via hormone exposure in utero. Treating rat dams with estrogen in late gestation results in greater mammary ductal growth in female offspring postnatally but also increased incidence of tumorigenesis [37]. Lower placental production of estrone sulfate, an estrogen precursor, has been reported in heat stressed animals [39] and the fetal mammary gland is receptive to estrogen [36]. Thus, reduced exposure of IUHT fetuses to estrogen could potentially be a contributing factor to retarded mammary growth. In addition, mammary stem cells are critical to mammary development. Progressive differentiation of stem cells leads to commitment to a specific cell type, such as ductal epithelial or epithelial alveolar cells [40]. Therefore, it is possible that alterations in early stem cell differentiation may induce aberrant mammary morphology.

Our data suggest that adverse conditions experienced in utero alters mammary morphology in a way that may compromise milk production in the first lactation. However, it is important to note that the majority of mammary growth occurs postnatally. Substantial ductal elongation and branching occurs in the peripubertal period and with each subsequent estrous cycle and extensive lobulo-alveolar development occurs during pregnancy [13]. Although IUHT and IUCL heifers were managed similarly after birth, effects of the intrauterine environment on the lactating mammary gland may still be partially confounded by the postnatal environment.

In summary, our data show that morphology of the lactating mammary gland of primiparous cows is altered by exposure to heat stress in utero, which may be associated with the observed phenotype of lower milk production. Deviations from the normal intrauterine environment appear to have consequences for the developing mammary gland that persist into adulthood, almost two years after the heat stress insult occurred. Further studies are needed to elucidate how in utero heat stress affects fetal and postnatal mammary development and to determine the specific mechanisms through which alterations in alveoli development affect milk production. Efforts aimed at improving production efficiency should consider how to modulate not only the ambient environment experienced by the dam but also the intrauterine environment experienced by the fetus.
